# Translating the Biology of Diffuse Large B-cell Lymphoma Into Treatment

**DOI:** 10.1093/oncolo/oyab004

**Published:** 2022-01-24

**Authors:** Alexey V Danilov, Massimo Magagnoli, Matthew J Matasar

**Affiliations:** City of Hope National Medical Center, Duarte, CA, USA; Humanitas Cancer Center, Humanitas Clinical and Research Center – IRCCS, Rozzano, Milan, Italy; Memorial Sloan Kettering Cancer Center, New York, NY, USA

**Keywords:** diffuse large B-cell lymphoma, biology, classification, cell of origin, precision medicine

## Abstract

Diffuse large B-cell lymphoma (DLBCL) is characterized by clinical and molecular heterogeneity; however, this heterogeneity is rarely taken into account by standard-of-care treatment approaches. While the disease was traditionally classified based on transcriptome signatures purporting the tumor cell of origin, recent classification systems have further differentiated these subtypes into clusters based on molecular and genetic features. Alongside a better understanding of the biology of the disease and the signaling pathways involved, emerging therapeutic agents may be better aimed at attacking distinct disease subsets. It is hoped that molecular subtyping at diagnosis will allow patients to be allocated to the appropriate treatment that targets their specific disease subtype, thus advancing the promise of precision medicine in lymphoma, an approach that is most needed. For high-risk disease subsets, this is particularly important, and much research is still needed to develop agents effective in this population. Here, we review recent advances in DLBCL biology and how they can be translated into clinical care.

## Teaser

This article reviews recent advances in the biology of diffuse large B cell lymphoma and how to translate into better management of the heterogenous disease.

**Implications for Practice**

Diffuse large B-cell lymphoma (DLBCL), the most common type of non-Hodgkin lymphoma, is a heterogenous disease with a high relapse rate and poor outcomes in the relapsed/refractory setting. Assessing the molecular profile is fundamental to the diagnosis but can also guide treatment decisions. Advanced understanding of DLBCL biology has facilitated the development of novel drugs in this area. Sophisticated classification methods that incorporate molecular characteristics and other prognostic indicators are likely to transform the management of DLBCL and improve the outcomes for patients.

## Introduction

Diffuse large B-cell lymphoma (DLBCL) is the most common type of non-Hodgkin lymphoma (NHL), accounting for around a third of all cases.^1^ The disease is both clinically and molecularly heterogenous, with distinct subtypes traditionally classified based on the cell of origin (COO) of the tumor. More recently, novel classification systems have described subtypes of DLBCL based on molecular and genetic signatures.^2-5^ The current standard of care for front-line treatment of DLBCL is usually R-CHOP.^6^ However, around 40% of patients will develop relapsed or refractory (R/R) disease, with poor subsequent outcomes.^7,8^ Considering the biological and clinical heterogeneity of DLBCL, and the need for more effective therapies, this review focuses on identifying possible avenues to translate this theoretical knowledge into clinical practice.

## Stratification of DLBCL

Most NHLs, including DLBCL, originate in the germinal centers (GC) of lymph nodes, which also give rise to trademark heterogeneity of DLBCL. GCs are specialized, transient structures that develop upon antigen challenge. Here, B-lymphocytes undergo maturation to become either plasma B cells, which secrete high-affinity antibodies, or memory B cells primed against future infection. The GC therefore facilitates T-cell-dependent humoral immunity and adaptive immunity.^9^ As B cells transit the GC, class switch recombination confers a risk of unintended mutations leading to lymphomagenesis.

### Prognostic Indices

Various clinical indices have been used with the goal of risk stratification in DLBCL, including the International Prognostic Index (IPI),^10^ revised IPI (R-IPI)^11^ and National Comprehensive Cancer Network IPI (NCCN-IPI).^12^ In addition, the CNS-IPI combines the prognostic factors comprising the IPI with renal and/or adrenal gland involvement in estimating the risk of developing secondary CNS relapse.^3^ This has been further refined with the inclusion of COO in the prognostic model.^13^

While these indices have utility in predicting outcomes, they do not routinely lead to a qualitative change in the chemotherapy backbone. By integrating molecular features into prognostic models, a resulting “molecular IPI” could potentially better characterize patients at high risk of failure with R-CHOP, for whom novel treatment approaches are most needed.^14^

### Cell of Origin and Prognostic Significance

Gene expression profiling (GEP) classifies DLBCL into biologic subtypes arising from distinct stages of normal B-cell development, which bear distinct genetic abnormalities and respond differently to chemoimmunotherapy (CIT) and targeted agents. The 2 COO-based subtypes include germinal center B-cell-like (GCB) and activated B-cell-like (ABC), GEPs typical of cells at these stages of differentiation.^15^ Later efforts described a third, “unclassified” or “type 3” subtype, which did not conform to either GCB or ABC DLBCL.^16^

GCB is the more prevalent subtype. One population-based study reports GCB in 56% of the cohort, with 32% ABC and 11% type 3.^17^ Studies conducted in the pre-rituximab era, showed significantly better outcomes for patients with GCB DLBCL than those with ABC subtypes (5-year overall survival [OS] rates: 60%-70% and 16%-35%, respectively). However, in the post-rituximab era, these figures have improved to 78% and 56%, respectively.^17^ In prospective trials, COO has had a more modest association with outcomes. In the GOYA trial, 5-year progression-free survival (PFS) rates for GCB, ABC, and type 3 were 66%, 56%, and 63%, respectively among patients receiving R-CHOP.^18,19^

**Figure 1.**
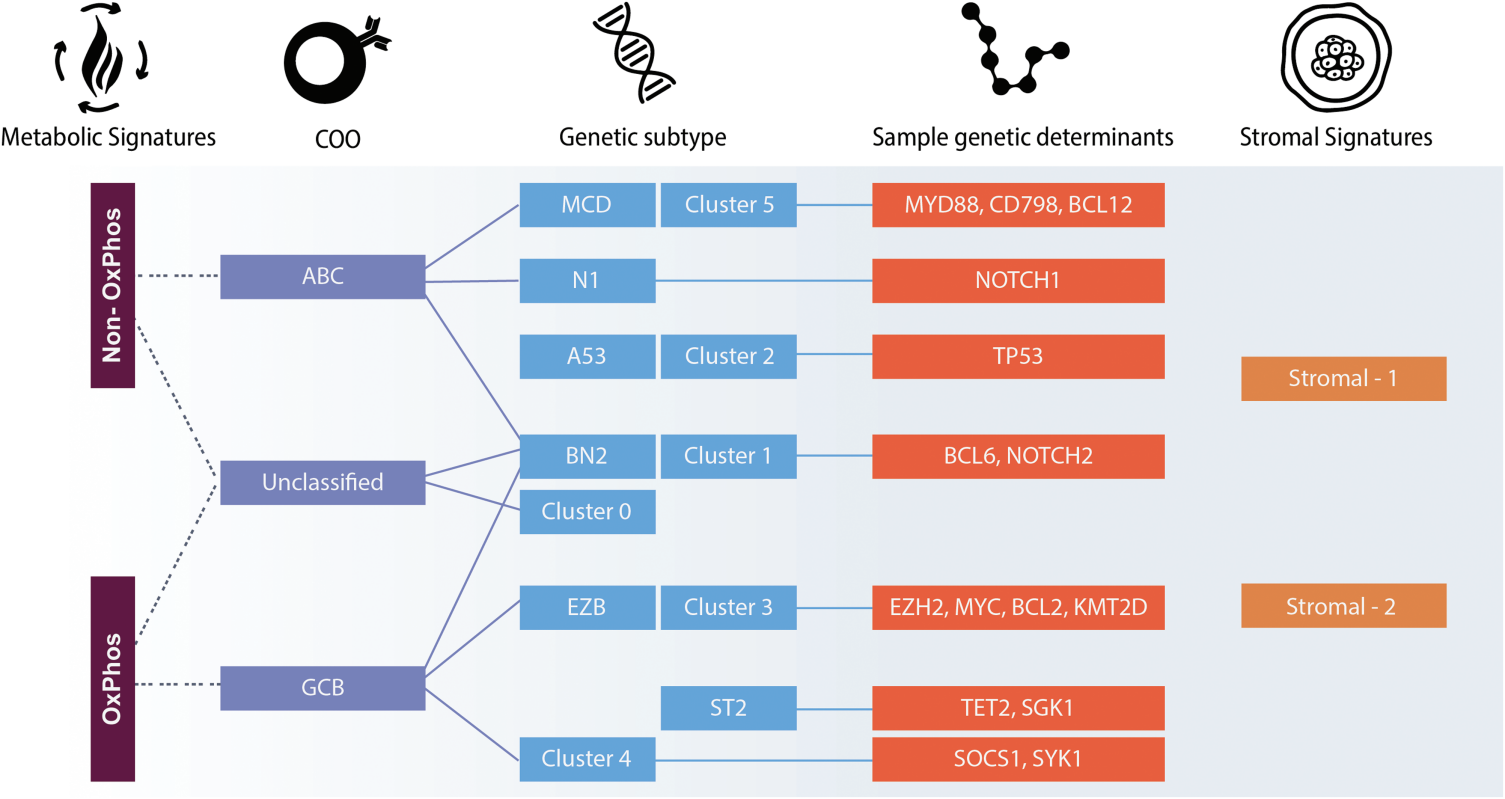
DLBCL subtypes and frequent genetic alterations. The figure depicts GEP-based classifications (COO, metabolic, stromal), the novel genetic subtype classifications and possible interface between them.^2-4,20,21^ Abbreviations: ABC, activated B-cell; COO, cell of origin; GCB, germinal centre B-cell; GEP, gene expression profiling.

Efforts also concentrated on alternative approaches to classify DLBCL. Caro et al relied on GEP to subdivide DLBCL based on the metabolic program, while Lenz et al analyzed stromal gene signatures to identify the prognostically favorable “stromal-1” and unfavorable “stromal-2” subtypes.^20,21^ How these DLBCL subsets interface with novel genetic classifications is unknown (Figure 1). Since GEP is not widely accessible in clinical practice, immunohistochemistry (IHC)-based approaches were introduced to distinguish between COO subtypes. The Hans algorithm, based upon expression of CD10, BCL6, and IRF4/MUM1, is used most.^22^ Although IHC-based approaches are rapid and cost-effective, they do not reliably identify the ABC subset, resulting in misclassification of cases at meaningful rates.^23^

Some DLBCL subgroups have distinct molecular profiles that are closely related to high-grade B-cell lymphoma or primary mediastinal B-cell lymphoma.^24-26^ These cases, together with T-cell/histiocyte-rich B-cell lymphoma, contribute to the “unclassified” COO and may be separated from DLBCL in future classifications.

### COO Subtypes Refined by Genetic Alterations

Genetic alterations in DLBCL determine B-cell signaling and differentiation stage, including chromosomal translocations, somatic mutations, and copy-number alterations.^27^ Two groups independently explored the genetic and mutational signatures in DLBCL.^2-4^ Using different integrated genomic approaches, they classified DLBCL cases into clusters featuring common genetic signatures, associated with outcomes. The feasibility of this type of testing in DLBCL was confirmed by other groups.^28,29^ These genetic subtypes and their common genetic alterations are shown in Figure 1. Based on these findings, a probabilistic algorithm was developed to determine the genetic subtype of an individual, allowing for prognostication and therapeutic decision-making, including defining candidates for precision medicine trials.^4^

Lymphomas with chromosomal rearrangements of *MYC* and *BCL2* and/or *BCL6* (double- and triple-hit lymphoma, now classified as high-grade lymphoma) are commonly of GCB subtype. Patients harboring these mutations have particularly poor outcomes when treated with R-CHOP, and while outcomes may vary according to the *MYC* translocation partner, intensified induction is considered appropriate by the majority.^30-35^ Such patients should be distinguished from those with overexpression (but lacking gene rearrangements) of MYC and BCL2 (double-expressor lymphoma), which typically are of ABC subtype.^2-4,20,21^ Although prognosis may also be inferior in these patients, intensified induction has demonstrated to improve outcomes. Similarly, mutations in *TP53* have been linked to acquired rituximab resistance and R-CHOP failure, and are enriched in R/R DLBCL.^36^

However, a unified model that is suitable for implementation in clinical practice remains to be delineated. The model used by Schmitz et al leaves a large proportion of cases as unclassified and relies on complex input, including exome sequencing and copy-number analysis, that is unlikely to be translated into clinical practice.^5^ Other classifications, such as the approach described by Lacy et al, rely on more practical multigene panels.^28^ Fundamentally, our improving understanding of molecular and genetic subtypes potentiate the rational investigation of incorporating targeted therapies into the treatment of DLBCL.

## The Evolving Treatment Landscape of DLBCL

### Current Standard of Care

R-CHOP remains the first-line standard of care in DLBCL. Attempts to escalate therapy or to introduce alternative anti-CD20 monoclonal antibodies (mAbs) to improve outcomes in the first-line setting have been largely unsuccessful in improving OS. The GOYA study (*N* = 1418), a head-to-head comparison of R-CHOP with the anti-CD20 mAb obinutuzumab combined with CHOP (G-CHOP), showed no survival benefit.^19,37^ A randomized cooperative group Phase 3 study investigating the increased dose-intensity regimen DA-EPOCH-R versus R-CHOP (*N* = 524) also demonstrated no benefit of therapy intensification.^38^ The only study to show improved OS in the front-line setting was an open-label randomized study using dose-intensive rituximab, doxorubicin, cyclophosphamide, vindesine, bleomycin, and prednisone in patients <60 years with low-intermediate risk IPI DLBCL (*N* = 379). Three-year OS was 92% versus 84% with R-CHOP (*P* = .0071).^39^

For patients with R/R disease, platinum-based salvage CIT followed by autologous stem cell transplant remains the standard of care for younger, fit patients.^40-42^ The management of transplant-ineligible patients, who comprise up to 60% of patients, is tailored to the individual patient’s tolerance; given poor outcomes, such patients represent a high unmet need.^43^

### Targeting Treatment by COO Subtype

A major focus of research in DLBCL has been to leverage our understanding of the distinct biology of DLBCL COO to deploy novel therapies. In the front-line setting, 4 large, randomized phase 3 studies have investigated whether outcomes can be improved by combining agents targeted to COO subtypes with standard R-CHOP therapy. Three failed to meet their primary endpoints (Table 1),^44-46^ including the ROBUST trial comparing R-CHOP with or without lenalidomide. A second randomized trial studying this combination, ECOG-ACRIN E1412, reported marginally positive findings (3-year PFS of 73% vs 61%, 1-sided *P* = .03, 3-year OS of 83% vs 75%, 1-sided *P* = .05).^47^ Additional phase 3 studies attempting to reduce the risk of relapse in COO-stratified patients who achieved remission with R-CHOP have also been largely unsuccessful.^48-50^ A recent press release indicates that the impending results of the placebo-controlled phase 3 POLARIX study (NCT03274492), studying Pola plus R-CHP versus R-CHOP in patients with previously untreated DLBCL, may be positive. The primary endpoint of improved PFS seems to have been met; at the time of writing, the results had not been published or presented.

**Table 1.** Phase 3 studies aimed at improving DLBCL outcomes with first-line R-CHOP.

Study	Patients (no. randomized)	Regimen	Endpoints/Response
R-CHOP combinations			
PHOENIX^44^ NCT01855750	Previously untreated non-GCB-DLBCL (*N* = 838). Initially selected by IHC; retrospective GEP analysis	R-CHOP +/– ibrutinib (RI-CHOP)	EFS: Not met in overall ITT or ABC population but significant interaction for RI-CHOP in patients <60 years (EFS [HR, 0.579], PFS [HR, 0.556], and OS [HR, 0.330]); ORR and CR were similar in overall ITT or ABC
ROBUST^45^ NCT02285062	Previously untreated, CD20+, ABC DLBCL (Lymph2Cx GEP) with Ann Arbor stage II-IV (*N* = 570)	R-CHOP +/– lenalidomide (R2-CHOP)	PFS: Not reached in either arm At median follow up of 27.1 months OS was 79% for R2-CHOP and 80% for R-CHOP; ORR 91% for both arms, CRs 69% for R2-CHOP and 65% for R-CHOP
REMoDL-B^46^ NCT01324596	Newly diagnosed DLBCL patients – stratified using GEP (*N* = 244 ABC, *N* = 475 non-GCB, *N* = 199 unclassified)	R-CHOP +/– bortezomib (RB-CHOP)	PFS: No difference between R-CHOP and RB-CHOP in GCB vs ABC DLBCL: 30-month PFS 70.1% (95% CI 65.0-74.7) vs 74.3% (69.3-78.7), respectively; HR 0.86 (0.65-1.13); *P* = .28
ECOG-ACRIN E1412^47^ NCT01856192	Newly diagnosed DLBCL patients (Lymph2Cx GEP: *N* = 49 ABC, *N* = 122 GCB, *N* = 64 unclassified)	R-CHOP +/– lenalidomide (R2-CHOP)	PFS: At median follow up of 3.0 years, R2CHOP vs R-CHOP showed a HR 0.66 (95% CI, 0.43 to 1.01). 3-year PFS was 73% vs 61% (*P* = .03) The PFS HR for R2CHOP was 0.67 for ABC-DLBCL (*P* = .1)
Maintenance therapy			
PRELUDE^48^ NCT01122472	Patients in complete remission after 6 or 8 cycles of R-CHOP and at high risk of relapse (*N* = 650)	Maintenance therapy with lenalidomide vs placebo	Significant difference in median PFS in favor of lenalidomide vs placebo in overall population (HR 0.708 [0.537-0.933]; *P* = .01) and in GCB patients (HR 0.491 [0.245-0.985]; *P* = .04) but not in ABC patients
REMARC^49^ NCT00332202	Patients in complete remission after 6 cycles of R-CHOP and at high risk of relapse (*N* = 215 with evaluable COO by Hans)	Maintenance therapy with enzastaurin vs placebo	DFS: No significant differences in DFS and OS between GCB (*n* = 109) and non-GCB (*n* = 106) subgroups (DFS, HR, 0.92 [0.56-1.52]; *P* = .742; OS HR, 0.72 [0.39-1.35]; *P* = .307) for enzastaurin vs placebo
PILLAR-2^50^ NCT00790036	Poor-risk patients who had achieved a CR with R-chemo (*N* = 742: *n* = 349 ABC, *n* = 264 non-GCB)	“Adjuvant” everolimus vs placebo for 1 year	DFS: No significant difference vs placebo in overall population (HR 0.92 (0.69-1.22); *P* = .276. Two-year DFS rate: 77.8% (72.7-82.1) with everolimus; 77.0% (72.1-81.1) with placebo DFS No significant differences in DFS or OS between GCB and non-GCB subgroups

Abbreviations: ABC, activated B-cell; COO, cell of origin; CR, complete response; DFS, disease-free survival; DLBCL, diffuse large B-cell lymphoma; EFS, event-free survival; GCB, germinal center B-cell; GEP, gene expression profiling; ORR, objective response rate; OS, overall survival; PFS, progression-free survival.

Further tailoring of trial populations based on genetic abnormalities seems to be a way to overcome the negative trend. A flexible trial design, allowing for addition of targeted agents later in the treatment course instead of at the very beginning, and narrowing screening windows may also help improve outcomes. Currently, clinical trials often select biologically more favorable patients; an approach that is closer to clinical practice is needed. Another solution would be to focus trials exclusively on a high-risk patient population, such as high IPI or double-hit lymphoma.

### Tractable Targets in GCB DLBCL

BCL2 is a key member of the family of proteins that mediate the apoptotic response to anticancer therapeutics.^51^ BCL2 overexpression is associated with poor prognosis.^52,53^ In the phase 1b portion of the CAVALLI study (NCT02055820), first-line treatment with venetoclax plus R-CHOP or G-CHOP demonstrated promising activity and safety.^54^ Particularly high efficacy (87.5% complete response [CR] rate) was observed in double expressor DLBCL, supporting further investigation in high-risk patients with BCL2+ DLBCL or DHL.^54^ The open-label phase 2 portion of the study (*N* = 206) showed promising efficacy of adding Bcl2 inhibitor venetoclax to first-line R-CHOP in 206 patients with DLBCL, including 101 patients with GCB DLBCL and 48 with the ABC subtype (69% CR rate with venetoclax in combination with R-CHOP), although caution is warranted, since this was not a randomized comparison.^55^ There was a trend toward improved PFS (HR = 0.61) with combination therapy versus R-CHOP alone, but this was offset by increased myelosuppression: 86% of patients who received Bcl-2 inhibitor had grade 3/4 hematologic adverse events, versus 66% with R-CHOP alone. A randomized phase 2/3 study is currently investigating first-line venetoclax combined with CIT in patients with *MYC/BCL2* double-hit and double expressing lymphomas (NCT03984448).

Dysregulation of epigenetic pathways is implicated in lymphomagenesis, and mutation or overexpression of histone-methyl transferases such as EZH2 (enhancer of zeste homolog 2) have been linked to the development of GCB-type DLBCL.^56^ Tazemetostat, a potent, orally available, selective small molecule inhibitor of EZH2 enzymatic activity, is under investigation in combination with R-CHOP (Epi-RCHOP) as a first-line treatment for newly diagnosed, poor prognosis DLBCL (NCT02889523).

*MYC*-directed approaches to targeting GCB DLBCL under investigation include bromodomain and CDK9 inhibitors. Bromodomain inhibitors exert antitumor activity largely by disrupting *MYC* transcription, which is regulated by BRD4, a member of the bromodomain and extra-terminal (BET) family of proteins.^57^ An oral BET inhibitor, CC-90010, is in early development in DLBCL having demonstrated single-agent activity in a phase 1 study in several solid tumors (NCT03220347).

Cyclin-dependent kinases (CDKs) are serine/threonine kinases that play key roles in cell cycle regulation and RNA transcription. The selective CDK9 inhibitor AZ4573 is in early clinical development in DLBCL (NCT03263637). Preclinical studies have demonstrated that selective targeting of CDK9 restricts the growth of DLBCL cells independent of COO, by halting transcription and downregulating MCL1 and MYC expression,^58^ and targeting CDK9 has been shown to disrupt MYC oncogenic activity in DLBCL. The novel CDK9 inhibitor A-1592668 combined with venetoclax demonstrated synergistic activity both in vitro in lymphoma cell lines and ex vivo in DLBCL biopsies.^59^ These findings suggest that dual inhibition of CDK9 and BCL2 may be effective in tumors reliant on overexpression of the anti-apoptotic BCL2 family proteins.

### Tractable Targets in ABC DLBCL

#### Front-line Options in Clinical Development

Neoplastic B cells rely on BCR signaling in their survival, however, unlike the GCB subtype, ABC DLBCL is dependent on constitutive activation of the nuclear factor kappa-B (NF-κB) signaling. Mutations in genes downstream of the BCR (eg, *CD79*, *MYD88, CARD11)*, and NF-κB-related genes are enriched in ABC DLBCL, resulting in chronic activation of BCR signaling, thus making components of this pathway attractive therapeutic targets.^60^ Several inhibitors of Bruton’s tyrosine kinase (BTK), a key kinase in BCR signaling, are under investigation in DLBCL. Ibrutinib is a potent covalent inhibitor of BTK.^61^ In the PHOENIX study (see Table 1 for details), OS was improved among younger patients (<60 years) treated with ibrutinib plus R-CHOP versus R-CHOP alone (NCT01855750).^44^ Acalabrutinib, a second-generation selective BTK inhibitor,^62^ is now under investigation in combination with R-CHOP in adult patients up to age 65 with newly diagnosed ABC DLBCL (ESCALADE; NCT04529772).

Proteasome-targeting agents abrogate constitutive NF-κB activity and enhance the pro-apoptotic effect of chemotherapy in DLBCL cells in vitro.^46,63^ Proteasome inhibitor bortezomib has shown higher efficacy in this subtype over GCB DLBCL in a phase 2 trial.^63^ However, the phase 3 REMoDL-B trial found no significant improvement in efficacy of adding bortezomib to R-CHOP in GCB or ABC DLBCL.^46^ In the front-line setting, bortezomib is under investigation in the phase 1/2 ImbruVeRCHOP study (NCT03129828) in combination with both R-CHOP and ibrutinib in newly diagnosed, high-risk (by IPI) DLBCL patients^64^

The PI3K-(phosphatidylinositol-3-kinase) AKT pathway plays a critical role in cell survival in response to extracellular signals.^65^ In the front-line setting, copanlisib, a pan-class I small molecule PI3K inhibitor with predominant activity against p110α and δ isoforms approved for R/R follicular lymphoma, is under investigation in a phase 2 study in combination with R-CHOP (Copa-R-CHOP; NCT04263584).^66^ While PI3K may be dysregulated in both GCB and ABC DLBCL, and loss of PTEN, a negative pathway regulator, is exclusive to GCB subtype,^67,68^ preclinical data suggest that copanlisib may be particularly efficacious in the ABC-DLBCL subset, where it may cooperate with BTK inhibition.^69^ In the R/R setting, single agent copanlisib has demonstrated activity in a phase 2 study in 67 R/R DLBCL patients stratified by COO and *CD79B* mutational status (NCT02391116). Objective response rates (ORR) of 31.6% with 21.1% CR, and 13.3% ORR with 3.3% CRs were observed in ABC and GCB patients, respectively. The ORR in patients with or without *CD79B* mutations was similar (22.2% vs 20%).^70^ The combination of copanlisib and the PD-1 inhibitor nivolumab is also under investigation (NCT03484819; NCT03884998).

Umbralisib (TGR-1202), a dual inhibitor of PI3K-δ and CK1ε (creatine kinase 1e), is being investigated in R/R DLBCL patients in the phase 2b UNITY-NHL study in combination with the anti-CD20 mAb ublituximab (NCT02793583).^71^

Preclinical evidence also provides a rationale for the combination of PI3K-inhibition with BCL2 blockade in ABC DLBCL.^69,72^ Copanlisib was found to induce apoptosis and modulate BCLX and MCL1 activity in BCR-dependent DLBCL cell lines with a genetic signature indicative of BCL2 dysregulation. This in vitro activity was synergistic when co-administered with the BCL2 inhibitor venetoclax, with this synergism also confirmed in a murine xenograft model.^72^ An ongoing phase 1/2 study investigates copanlisib in combination with venetoclax in patients with R/R DLBCL (NCT04572763).

#### Clinical Development in Relapsed/Refractory Setting

A recent meta-analysis has confirmed that ibrutinib-containing therapy can improve outcomes in patients with non-GCB DLBCL.^73^ Single-agent ibrutinib has shown promising activity in R/R primary and secondary CNS lymphoma (PCNSL/SCNSL), a uniformly ABC-type disease. In a phase 1 trial, 10/13 (77%) PCNSL patients responded to high-dose ibrutinib (840 mg), 5 with a CR and 5 with a partial response (PR).^74^ More recently, ibrutinib in combination with methotrexate and rituximab has shown to be well tolerated, with an 80% response rate (*N* = 12/15) in R/R CNS lymphoma (NCT02315326).^75^

In the phase 1/2 BRUIN trial, pirtobrutinib (LOXO-305), a noncovalent BTK inhibitor, showed activity in patients with B-cell malignancies (*N* = 323), including patients previously treated with covalent BTK inhibitors (NCT03740529).^76^ Noncovalent BTK inhibitor ARQ 531 is also under investigation in a phase 2 trial including patients with selected hematologic malignancies (NCT03162536).^77^

The immunomodulatory drug lenalidomide was studied in patients with DLBCL who had received ≥2 prior therapies, compared with investigator-choice chemotherapy. Lenalidomide modestly improved ORR (27.5% vs 11.8%) and PFS (13.6 vs 7.9 weeks), with greater improvements observed in non-GCB patients.^78^ Following the inconclusive results from ROBUST (Table 1),^45^ R-CHOP plus or minus lenalidomide is currently being investigated in newly diagnosed double-expressor DLBCL (NCT04164368).

Lenalidomide has also been successfully combined with tafasitamab (MOR208), an Fc-engineered antibody that targets CD19. In a phase 2 study, the combination showed encouraging activity in R/R DLBCL patients ineligible for high-dose chemotherapy and autologous stem cell transplant (L-MIND; NCT02399085), with an ORR of 60% and CR rate of 43%.^79^ The combination is now FDA approved for the treatment of R/R DLBCL.

### COO Agnostic Therapies

#### Antibody Drug Conjugates

Antibody drug conjugates (ADCs), which combine a cytotoxin with a target-specific mAb via a linker, are a promising treatment modality for DLBCL. The first-in-class ADC in DLBCL, polatuzumab vedotin (Pola), targets CD79b, a component of the B-cell receptor. It was granted accelerated FDA approval in 2019 in combination with bendamustine and rituximab (Pola-BR) for the treatment of patients with R/R DLBCL who have received ≥2 prior therapies, and there are European and Asian regulatory approvals for R/R DLBCL in the second line and beyond. Approval was based on a randomized phase 1b/2 study demonstrating an improved CR rate (40% with Pola-BR vs 18% with BR), and OS (12.4 vs 4.7 months, respectively).^80^ Pola-BR appeared to benefit patients regardless of COO, with a slight trend toward enhanced efficacy in ABC. CR rates were 47% and 27% in ABC- and GCB-patients, respectively. The HR versus placebo for PFS was 0.20 (95% confidence interval [CI], 0.09-0.45) for ABC and 0.49 (95% CI, 0.23-1.05) for GCB.^80^ Early findings in front line have also been promising when used in combination with R-CHOP minus vincristine (R-CHP). As discussed above, the phase 3 trial POLARIX, comparing R-CHP versus Pola plus R-CHOP, has completed accrual with first results anticipated in 2021 (NCT03274492). Two other phase 3 studies are underway in R/R DLBCL, evaluating platinum-based CIT with or without Pola. POLARGO (NCT04182204) is assessing rituximab plus gemcitabine plus oxaliplatin (RGemOx) with or without Pola, while PolaR-ICE (NCT04665765) is evaluating Pola with rituximab and ifosfamide-carboplatin-etoposide (RICE) and post-transplant Pola consolidation, in transplant-ineligible and eligible patients, respectively.

Emerging ADCs include loncastuximab tesirine (ADCT-402), an anti-CD19 antibody conjugated to a pyrrolobenzodiazepine dimer toxin.^81^ It is now in phase 2 of development as a single agent in R/R DLBCL after promising phase 1 findings (NCT03589469). Interim phase 2 study results presented at EHA 2020 demonstrated an ORR of 45.5% (20% CR). A phase 2 trial in the R/R setting is also underway in combination with ibrutinib (NCT03684694).

#### CART-cell Therapy

Chimeric antigen receptor-modified (CAR) T cells are presumed independent of COO subtype (reviewed by Hopfinger et al^82^). Two CD19-targeting CART products have now been approved in R/R DLBCL: axicabtagen ciloleucel (axi-cel) and tisagenlecleucel. Approvals were based on pivotal trials with ORRs of 82% (ZUMA-1; NCT02348216) and 52% (JULIET; NCT02445248). In ZUMA-1, of 74 patients assessed for COO, 52 (70%) were GCB-type and 18 (24%) ABC-type; COO was not associated with outcomes.^82,83^ Similarly, in JULIET (57% GCB; 41% non-GCB;), outcomes were not associated with COO.^84^ Ongoing trials evaluating CART versus platinum-based second-line therapy with planned auto transplant include ZUMA-7 (NCT03391466), BELINDA (NCT03570892), and TRANSCEND (NCT02631044). Although clearly promising agents, access to CART is limited by a lengthy manufacturing process (2-4 weeks), reliance upon apheresis of the patient’s circulating T cells, availability of specialized resources, and cost.^85^ Furthermore. CART-cell therapy has been shown to have inferior outcomes among patients with significant medical comorbidities, representing a significant proportion of patients with R/R DLBCL.^86^

#### Immune Checkpoint Inhibitors

Tumors often exploit immune checkpoint pathways to evade elimination by the host immune system. Immune checkpoint molecule programmed death ligand 1 (PD-L1) interacts with its receptor PD-1 on T cells to suppress anti-tumor activity. In DLBCL, several *PD-L1* gene alterations, mostly translocations and amplifications, have been associated with response to PD-1 blockade.^87^ Patients with *PD-L1* alterations were also shown to have an inferior response to front-line CIT, making PD-1 blockade an attractive treatment option.^87^ Both overexpression of PD-L1 and *PD-L1* alterations are more common in non-GCB DLBCL but can be seen in all subtypes.^88,89^

A phase 1 study of single-agent nivolumab in patients with R/R DLBCL showed an ORR of 36%^90^ but results of a subsequent larger study were disappointing.^91^ In the front-line setting, the PD-L1 inhibitor durvalumab in combination with R-CHOP was safe; encouraging response rates were observed in patients with high-risk DLBCL, including double-hit lymphoma (NCT03003520). A recent feasibility study in treatment-naive DLBCL patients (*n* = 30) using the PD-1 inhibitor pembrolizumab combined with R-CHOP reported an ORR of 90% and CR of 77%.^92^ Pembrolizumab is also under investigation in a phase 1/2 study in combination with MK-4280, another PD-L1 inhibitor, which antagonizes the lymphocyte activation gene-3 protein, in patients with Hodgkin’s lymphoma and NHL (NCT03598608).

#### Bispecific Antibodies

Bispecific antibodies target a tumor antigen and engage immune function by co-targeting antigens on host immune cells. Blinatumomab, which binds to CD19 and CD3, showed a median PFS of 3.7 months and a median OS of 5.0 months, with an ORR of 43% (19% CR) in R/R DLBCL; median duration of response was 11.6 months (NCT01741792).^93^ Preliminary results from an open-label phase 2 study of single-agent blinatumomab after front-line R-CHOP reported an 89% ORR in patients with newly diagnosed, high-risk DLBCL (NCT03023878).^94^ Blinatumomab is also under investigation in R/R DLBCL in combination pembrolizumab (NCT03340766).

Bispecific antibodies mosunetuzumab and glofitamab co-target CD3/CD20. Durable CRs with mosunetuzumab, including in patients who had relapsed after CART-cell therapy, have recently been reported in a phase 1 study (ORR 39%, 22% CRs) (NCT02500407).^95^ Glofitamab as a single agent or in combination with obinutuzumab is under phase 1 investigation in R/R NHL (NCT03075696). Data on single-agent glofitamab with obinutuzumab pretreatment have been encouraging, with durable responses at the recommended phase 2 dose (ORR 41.4% and CR 28.8% in 73 patients with R/R DLCBL).^96^ Two other studies of glofitamab plus chemotherapy have been initiated: a phase 1 study of glofitamab plus R-CHOP in the front-line setting (NCT03467373) and a phase 3 study of glofitamab plus gemcitabine/oxaliplatin versus rituximab plus gemcitabine/oxaliplatin in the R/R setting (NCT04408638).

#### CD47 Blockade

CD47 is an antiphagocytic signal overexpressed by tumor cells to facilitate evasion from phagocytosis. Overexpression of CD47 is an independent predictor of poor prognosis in patients. In the dose-escalation phase of a phase 1b/2 trial, the first-in-class anti-CD47 antibody magrolimab (Hu5F9-G4) demonstrated promising efficacy in R/R DLBCL, with ORR and CR rates of 40% and 33%, respectively^97^; the expansion stage/phase 2 of the trial in combination with rituximab, is ongoing (NCT02953509), as are evaluations with other agents targeting CD47 such as TTI-622 (NCT03530683).

CD47 is a ligand for signal regulatory protein α (SIRPα), expressed by macrophages. Binding of CD47 to SIRPα stops macrophages from phagocytosis of tumor cells.^98^ A number of SIRPα inhibitors are currently in development.^99^

#### XPO1 Inhibition

Exportin 1 (XPO1, also known as CRM1), is a nuclear exporter protein that mediates the export of tumor suppressor proteins, including p53, out of the nucleus. Inhibition of XPO1 retains these suppressor proteins within the nucleus, restoring their tumor-suppressor function.^100^ Selinexor, an orally administered selective inhibitor of XPO1, was recently granted accelerated FDA approval based on the phase 2b SADAL study conducted in R/R DLBCL patients (NCT02227251; *N* = 127). The reported ORR was 28%, with 34% the GCB and 21% in the non-GCB subtype, although an unfavorable toxicity profile and strict inclusion/exclusion criteria may limit clinical applicability of these findings.^101^

## Future Directions

**Figure 2.**
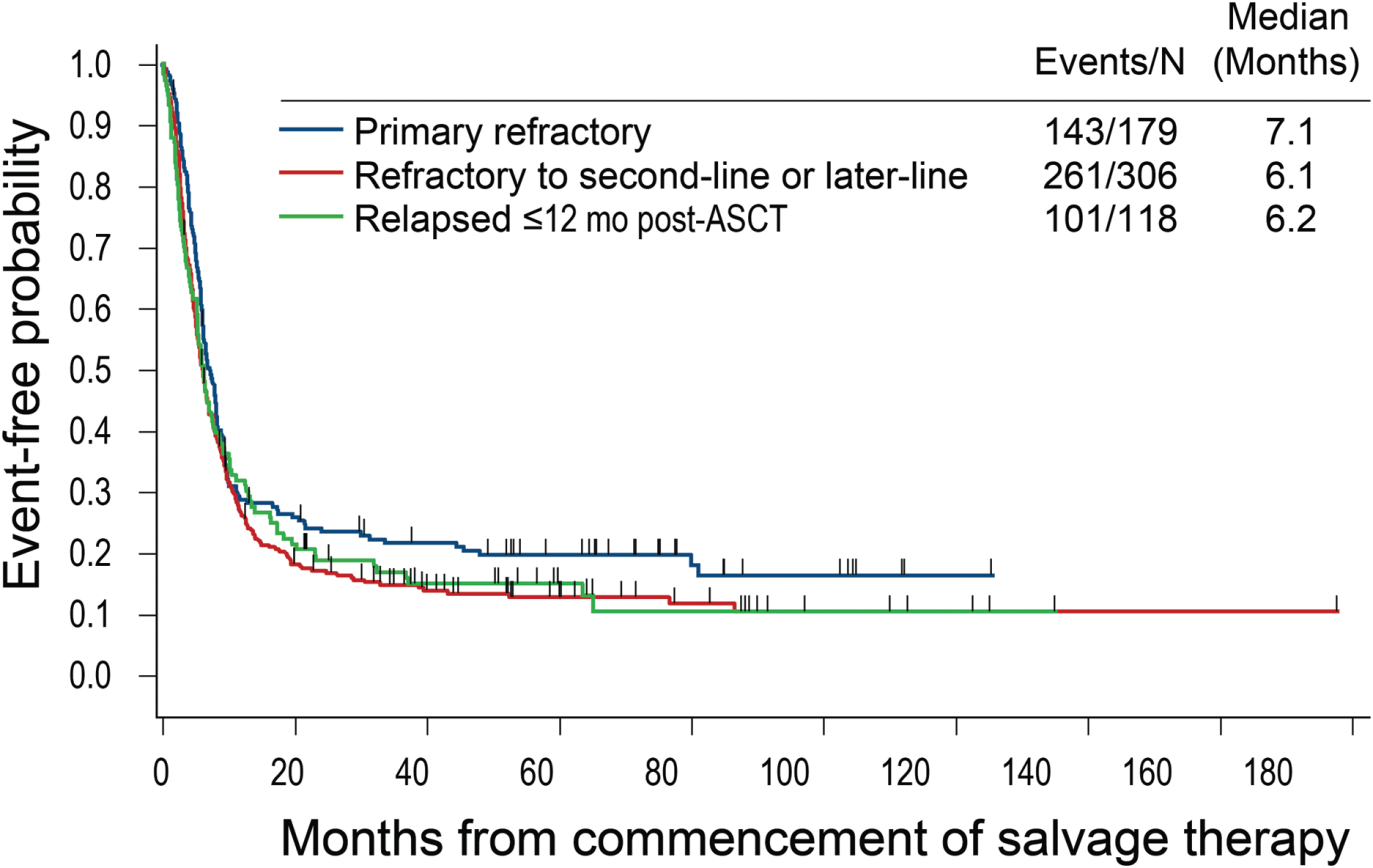
SCHOLAR-1 study: Overall survival on salvage therapy in refractory DLBCL patients^102^. Republished with permission of Elsevier Science & Technology Journals, from Outcomes in refractory diffuse large B-cell lymphoma: results from the international SCHOLAR-1 study, Crump M, et al, Blood. 2017;130(16):1800-8. Year of copyright: 2021; permission conveyed through Copyright Clearance Center, Inc. SCHOLAR-1 is an international, multicohort research study that retrospectively evaluated outcomes in patients with refractory DLBCL, defined as progressive disease or stable disease as best response at any point during chemotherapy (>4 cycles of first-line or 2 cycles of later-line therapy) or relapsed at ≤12 months from autologous stem cell transplantation. SCHOLAR-1 pooled data from two phase 3 clinical trials (Lymphoma Academic Research Organization-CORAL and Canadian Cancer Trials Group LY.12) and two observational cohorts (MD Anderson Cancer Center and University of Iowa/Mayo Clinic Lymphoma Specialized Program of Research Excellence). Abbreviations: ASCT, autologous stem cell transplantation; mo, months.

The prognosis and treatment of patients with DLBCL depends on vast clinical and molecular heterogeneity. Assessing the molecular profile is fundamental to the diagnosis, including IHC for expression of MYC, BCL2 and BCL6 and fluorescence in situ hybridization for *MYC* and *BCL2* rearrangements. The SCHOLAR-1 study highlighted the futility of chemotherapy approaches in patients with DLBCL who are refractory to first-line CIT or relapse after 2 lines of chemotherapy (Figure 2).^102^ Hence there is an urgent medical need to improve the current standard of care for DLBCL, especially for molecularly defined subgroups at particularly high risk to exhibit resistance to first-line CIT. With advanced understanding of its biology, further parsing of DLBCL is poised to facilitate the development of novel agents for patients with specific needs. Concurrent development of sophisticated classification methods that incorporate molecular characteristics and other prognostic indicators is likely to transform the management of DLBCL and improve the outcomes for patients with high-risk disease. While current prognostic indices can aid in predicting outcomes, more accurate indices that incorporate both COO classification and/or genetic signatures would be more likely to guide treatment choice.

Several large phase 3 trials in the front-line setting have attempted to improve outcomes by combining novel targeted agents with standard of care CIT regimens, but these have been largely unsuccessful. It remains to be seen whether these failures were due to bias, such as including low-risk patients who would be adequately treated with standard of care could have unwittingly masked any favorable efficacy in high-risk patients. This begs the important question as to whether all-comer trials are still appropriate in DLBCL. Emerging data raise expectations for an increasing role of genetic profiling in DLBCL, with hopes that it will soon follow in the steps of myeloid leukemia (“Beat DLBCL”). Informed and refined by novel classifications, prospective trials in the front-line setting might perform molecular subtyping during the initial cycle of R-CHOP and then allocate patients to treatment with an appropriate targeted agent, hence translating biology into individualized treatment. We have great optimism that ongoing combination clinical trials will translate to improved outcomes for our patients in the near future.

## Data Availability

No new data were generated or analyzed in support of this research.
